# Analysis of Brain Functions in Men with Prostate Cancer under Androgen Deprivation Therapy: A One-Year Longitudinal Study

**DOI:** 10.3390/life11030227

**Published:** 2021-03-10

**Authors:** Vanessa Sánchez-Martínez, Cristina Buigues, Rut Navarro-Martínez, Laura García-Villodre, Noura Jeghalef, María Serrano-Carrascosa, José Rubio-Briones, Omar Cauli

**Affiliations:** 1 Department of Nursing, University of Valencia, 46010 Valencia, Spain; Vanessa.sanchez@uv.es (V.S.-M.); cristina.buigues@uv.es (C.B.); Rut.Navarro@uv.es (R.N.-M.); garcia_lauvil@gva.es (L.G.-V.); noujeg@alumni.uv.es (N.J.); 2 Frailty and Cognitive Impairment Research Group (FROG), University of Valencia, 46010 Valencia, Spain; 3 Haematology Department, Hospital General Universitario, 46014 Valencia, Spain; 4 Department of Urology, Fundación IVO, 46009 Valencia, Spain; marietadelao77@gmail.com (M.S.-C.); jrubio@fivo.org (J.R.-B.)

**Keywords:** neurotoxicity, testosterone, androgen-deprivation therapy, cognitive function, sleep, depression

## Abstract

The relationship between cognitive decline and androgen deprivation therapy (ADT) under luteinizing hormone-releasing hormone (LHRH) analogues is unclear, and there is a scarcity of longitudinal studies considering the interaction between cognition, depressive symptoms and sleep quality in men with prostate cancer (PCa) treated with ADT. This study aimed to determine if there were differences in the scores obtained in cognitive assessment, depressive symptoms, and sleep quality after one year of ADT and determine the interrelations between sleep, mood, and cognitive status. A prospective longitudinal observational study was designed, in which a cohort of men (mean age was 70.8 years) newly treated with androgen-deprivation therapy was assessed in the first six months of treatment and 12 months later. Analysis of cognitive function by the Mini-Mental State Examination (MMSE) scores indicated a significant (*p* < 0.05) increase after one year of treatment and by the Brief Scale for Cognitive Evaluation (BCog) scores indicated no changes in the scores before and after one year of treatment. Analysis of depressive symptoms with the Geriatric Depression Scale and sleep quality with the Athens Insomnia Scale (AIS) scores showed significant (*p* < 0.05) changes after one year of treatment with ADT, with men describing more depressive symptoms and more sleep disturbances. No statistically significant differences were found in the cognitive performance between men with impaired sleep or depression results and those without them. Our study showed no clinical evidence of the relationship between ADT under luteinizing hormone-releasing hormone (LHRH) analogues and cognitive deterioration in 1-year follow-up, but there are impairments in the sleep quality in men with PCa undergoing ADT and an increase in depressive symptoms which has important implications for clinicians as they would impair quality of life and adherence to treatment.

## 1. Introduction

Prostate cancer (PCa) is the second most frequent cancer in men [1]. In 2017, its global estimated incidence was 1.3 million, and it caused 416,000 deaths [2], with marked differences in the rates across different regions and populations [1,3]. In Europe, it was estimated to represent 21.8% of the total cancer incidence and 10% of cancer deaths in 2018 [4]. The diagnosis of cancer is a stressful experience that significantly impacts all spheres of patients’ lives, not only at the time of diagnosis but can be maintained for many years, even in those patients who have overcome the disease. However, not all sequelae in the cognitive-emotional sphere are produced by the impact of the diagnosis and the associated psychological disorders. In recent years, increasing importance has been given to the toxicity produced by oncology treatments, whether acute or late-onset. This stands out, especially the appearance of a cognitive deterioration associated with the administration of oncological treatments [5,6,7]. PCa is an androgen-dependent disorder, so the standard treatment is based on hormonal therapy to reduce the production of hormones that enhance tumour growth, mainly androgen deprivation therapy (ADT), in the form of chemical castration [8,9] and other antiandrogens. However, it is not exempt from numerous and often debilitating physical and psychological adverse effects that may affect the quality of life [10,11,12]. These can be classified into nine groups: musculoskeletal changes, metabolic changes, cardiac disorders, nervous system disorders, vascular disorders, hepatobiliary disorders, reproductive system disorders, psychiatric disorders, and general disorders [11].

Cognitive symptoms, depression and sleep disturbances are considered particularly challenging side effects of ADT [13]. More than a decade ago, some reviews suggested that treatment with androgen deprivation therapy in men with PCa could lead to subtle cognitive decline [14,15]. Some studies reported declines in different cognitive domains, such as verbal memory, executive function, spatial memory or visuomotor skills, while others failed to demonstrate a relationship between cognition and ADT [16]. It has been argued that the adverse effects of ADT could be negatively influenced by factors such as older age and lower education level [17]. However, despite subsequent studies, reviews and meta-analyses, there is no accepted consensus that this connection actually exists, as reviews show conflicting results [16,17,18,19,20,21,22] and the analysis of cognitive functions under ADT with different psychometric tools and the comparisons of changes in different cognitive domains under ADT is necessary in order to tailor interventions to minimise the ADT-induced toxicity effects upon brain function over time.

Cognitive impairment could also be associated with other known psychiatric adverse effects of ADT in men with PCa, such as depression [22,23,24] and reduced sleep quality [25,26]. Depression has been documented to increase in men with PCa, with a prevalence between 10% and 40% [27,28] that might be related to multiple factors, such as age, comorbidities, socioeconomic status, erectile dysfunction or the disorder itself [28,29,30]. Treatment with ADT has been associated with a higher increase of the incidence of depression in this group [23,28], which could impact not only cognition and quality of life but also on PCa prognosis [22,28,31]. Moreover, depression could be underdiagnosed and so, undertreated [29]; this is more relevant considering that one of its outcomes is the risk of suicide, also described to be increased in men with PCa [28,32]. It is accepted that insomnia symptoms are frequently aggravated by cancer treatments and their side effects [33,34], but there is scarce evidence of the relationship between ADT and sleep disturbances. Some studies have concluded that poor sleep quality appears in approximately one-third of the men treated with ADT, but the underlying physiological mechanism is unclear [35,36]. Among other factors, it has been related to hot flashes, nicturia and emotional distress and to the pharmacological treatment of these adverse effects [26,37,38,39].

Cognitive decline, mood disorders and poor sleep quality are adverse effects that are not easily attributable to one root cause. To summarise the literature gaps, the link between cognitive decline and ADT is unclear, and there is a scarcity of longitudinal studies considering the interaction between cognition, depressive symptoms and sleep quality in men with PCa treated with ADT. For many clinical research types, such as the psycho-geriatric evaluation parameters under chronic pharmacological treatment, longitudinal studies provide a unique insight into variables’ interactions that might not be possible in cross-sectional studies. They are beneficial when studying development lifespan issues such as cognitive function and associated factors.

In this context, this study aimed to study a cohort of men newly treated with ADT: to determine if, after one year of androgen deprivation therapy, there were differences between the baseline and the follow-up scores of the cognitive assessments; to measure the influence of these treatments in the mood and sleep quality; and to determine the interrelations between sleep, mood, cognitive status and other sociodemographic variables.

## 2. Methods

This is a prospective longitudinal observational study, in which a cohort of men newly treated with androgen-deprivation therapy with luteinizing hormone-releasing hormone (LHRH) analogues was assessed in the six months- to one year of treatment with LHRH analogues and at follow-up which was 12 months later (from the first evaluation). The trial was carried out in compliance with the guidelines of the Declaration of Helsinki, and the study protocol was approved by the local Ethics Committee (University of Valencia, Reference number: H1511682610849). All participants gave written informed consent before being enrolled in the study.

The participants were consecutively selected from an outpatient’s oncology practice if they fulfilled the eligibility criteria. Data collection was made between January 2018 and March 2020. Men were included in the first six months of treatment with long-acting injectable androgen deprivation therapy base don LHRH analogue (leuprorelin or triptorelin) and if they agreed to participate by signing the informed consent form. Men could not participate if they were receiving any other chemotherapy treatment for prostate or any other cancer, or if they had any known cognitive deterioration due to other causes. We excluded all those men who had completed the baseline assessment and suffered any relevant change in their health status that could influence their sleep quality, mood, or cognitive performance.

### 2.1. Sample Size

The sample size was determined before the development of the study, so the statistical power was calculated for the main outcome of the study which is cognitive function. Accepting an alpha risk of 0.1 in a one-sided test with 33 subjects in the first group and 33 in the second, the statistical power was 90% to recognise as statistically significant the difference from 0.09 in the first group to 0.34 in the second group.

### 2.2. Sociodemographic and Clinical Variables

The sociodemographic variables considered were: age (both numerical and categorised into men younger and older than 75 years); education level (classified into four groups, according to the maximum education level completed: no studies, primary studies completed (until 14 years of age), secondary school or vocational studies, university degree); marital status; employment status; the form of cohabitation. The clinical variables were metastases; previous prostatectomy; ADT treatment (the LHRH analogues triptorelin or leuprorelin).

### 2.3. Outcome Variables

The Mini-Mental State Examination (MMSE) score was considered as a numerical and categorical variable: normal or impaired. Besides, the scores of its dimensions (orientation, spatial orientation, immediate recall, attention and calculation, delayed recall, language) were considered independently as numeric variables. The Brief Scale for Cognitive Evaluation (BCog) score and its dimensions (communication, attention, recent memory, concentration, remote memory, orientation, calculation and executive function) were considered numerical. The Athens Insomnia Scale (AIS) score (numeric and categorised into normal or impaired). The Geriatric Depression Scale (GDS) score (numeric and categorised into normal or impaired).

### 2.4. Psychological Assessments

The cognitive status was evaluated through two different brief cognitive assessments: the BCog and the MMSE. The short version of the GDS was used as a screening for depression, and the presence of insomnia was measured through the AIS.

The MMSE is a brief cognitive test widely used [40] since it was created in 1975 by Folstein et al. [41] and validated into Spanish by Lobo et al. in 1999 [42] with adequate psychometric properties. It comprises 30 items grouped into six dimensions: orientation, spatial orientation, registration, attention and calculation, recall and language. The test can be completed in five to 15 min, and it has two cut-off points, depending on the age of the person assessed.

The BCog is a short cognitive battery recently validated in Spain for the general population and people with schizophrenia [43]. It comprises eight dimensions (communication, attention, concentration, short and long-term memory, orientation, calculation and information processing) and can be completed in less than 15 min. Its internal consistency, calculated through Cronbach’s alpha was 0.7 and its validity against the correlation with another brief cognitive test (Screening for Cognitive Impairment in Psychiatry), was 0.8.

The AIS is a self-report questionnaire used as a screening for sleep disturbances. Based on the International Classification of Diseases (ICD-10), the full scale is composed of eight items, with a score range from 0 to 24 (the cut-off point is six, and higher scores suggest a more serious problem) and it was validated by Soldatos et al. in 2000 [44]. The scale was validated into Spanish by Gómez-Benito et al. in 2011 [45] with acceptable psychometric properties (Cronbach’s alpha was 0.86).

The abbreviated version of the GDS is a screening questionnaire developed by Yesavage et al. [46]. It is composed of 15 items, and it was validated into Spanish in 2002 by Martínez de la Iglesia et al. [47] with acceptable psychometric properties and with a cut-off point of five or more.

### 2.5. Statistical Analysis

Descriptive statistical analyses were carried out. Kolmogorov–Smirnov tests were made to determine which variables adapted to the normal distribution. Only the baseline and follow-up BCog scores and those of two of its subtests (calculation and remote memory) adapted the normal distribution, so most of the statistical analyses carried out were non-parametrical. Bivariate correlations were calculated, both parametrical (Pearson’s) and non-parametrical (Spearman’s). Partial correlations were calculated to control the influence of the age and education level in the outcome variables. T-tests, one-way analyses of variance (ANOVAs), Mann–Whitney’s U tests and Kruskal–Wallis tests were carried out to determine if the differences between the values of the categorical variables were statistically significant. All the statistical tests were considered statistically significant at the level *p* < 0.05. The analyses were carried out using the IBM Statistical Package for Social Sciences SPSS (version 26.0; SPSS, Inc., Chicago, IL, USA).

## 3. Results

### 3.1. Characteristics of the Study Population

A sample of 44 men participated in the baseline assessment. Of them, 33 underwent the second assessment, so their results were analysed in this study. The reasons not to be reassessed were: three men refused to be screened for the second time, and eight suffered a health deterioration (for metastases requiring additional chemotherapy treatment or other reasons, such as cerebrovascular accidents) that could bias the results.

The mean age of the participants was 70.8 years, 13 (39.4%) men had completed compulsory education only (until 14 years of age), 27 (81.8%) were retired, 29 (87.9%) were married, and 24 (72.7%) lived only with their spouses. A total of 7 men had metastatic cancer, and 22 (66.7%) participants had previously a radical prostatectomy. Among men below age 75 years old, 71.4% had prostatectomy whereas among those aged 75 and over, 58.3% had prostatectomy. No significant differences were observed between prostatectomy and age group (*p* = 0.44, Chi-squared test). In the study sample, 11 patients were not submitted to a prostatectomy because 7 of them had bone-metastatic disease at diagnosis and 4 men received prostate brachytherapy as the main primary therapy. They were about to start or had started treatment with leuprorelin (9 men, 27.3%) or triptorelin (24 men) in the six months previous to the baseline assessment. Sociodemographic and clinical data are provided in more detail in Table 1.

### 3.2. Cognitive Evaluation, Depressive Symptoms and Insomnia Assessment in the Study Sample

The cognitive assessments showed different results (Table 2). On the one hand, the MMSE scores indicated a statistically significant increase after one year of treatment. On the other hand, the BCog scores indicated no statistically significant change in the scores before and after one year of treatment. The results obtained in the two assessments of the two cognitive tests applied were sensitive to the participants’ age and education level, as the differences obtained in the scores were statistically different (all *p* < 0.05). The scores were statistically different in the two groups in the two assessments, being higher for the youngest men (for the MMSE *p* < 0.05, and the BCog *p* < 0.001) and the most educated group (for the MMSE *p* < 0.05, and the BCog *p* < 0.01). The changes in baseline and follow-up scores of the MMSE and the BCog were compared in the two age groups (under 75 years old and 75 or more). For the MMSE, an increase was found in the two age groups, being statistically significant for the oldest group (*p* = 0.046), but not statistically significant for the youngest (*p* = 0.21). The results obtained in the BCog test showed an increase in the youngest group (*p* < 0.001), but a non-statistically significant reduction in the oldest (*p* = 0.79). We also considered the subtraction (difference in the scores obtained in the baseline and follow-up) for the two cognitive assessments in the two age groups, but these differences were not statistically significant for any assessment. By categorizing the age of the participants at 65 years old, we observed again a significant worsening of sleep quality during follow-up (*p* = 0.004) and no significant differences for other parameters.

**Figure 1:**
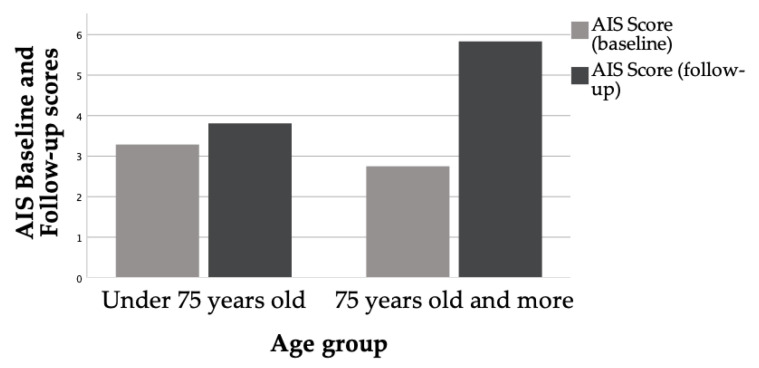
Evaluation of sleep quality at baseline and the follow-up according to age groups. AIS: Athens Insomnia Scale.

**Figure 2:**
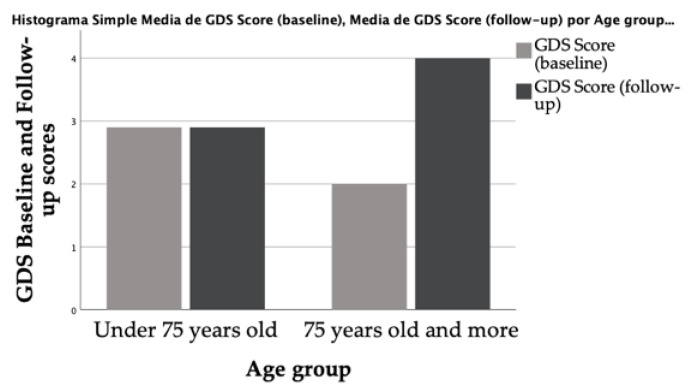
Evaluation of depressive symptoms at baseline and the follow-up according to age groups. GDS: Geriatric Depression Scale.

The GDS and the AIS scores showed changes after one year of treatment with ADT, with men describing more depressive symptoms and more sleep disturbances. However, only the differences in the AIS were statistically significant (for the AIS *p* = 0.018; for the GDS *p* = 0.194). Detailed information is offered in Figure 1 and Figure 2, and Table 2. The analysis considering age groups showed statistically significant differences for the AIS scores in the oldest group (*p* = 0.009).

### 3.3. Association between Cognitive Evaluation, Depressive Symptoms and Insomnia

The participants obtained different scores in the cognitive tests according to their academic level. These differences were statistically significant for the total scores of the two tests and several of their subtests, with men who had higher academic levels obtaining higher scores. The other categorical variables (marital status, the form of cohabitation, working status, previous prostatectomy, presence of metastasis or ADT drug) did not relate to statistically significant differences in the total nor partial scores of the cognitive tests. There was not significant association between cognitive function at baseline or at follow-up and PSA level (*p* = 0.63 and *p* = 0.64 for MMSE scale; *p* = 0.48 in both cases for BCog scale). There was not significant association between cognitive function at baseline or at follow-up and Gleason score (*p* = 0.27 and *p* = 0.47 for MMSE scale; *p* = 0.24 and *p* = 0.29 for BCog scale). There was not significant association between cognitive function at baseline or at follow-up and TNM stages (*p* = 0.45 and *p* = 0.46 for MMSE scale; *p* = 0.22 and *p* = 0.23 for BCog scale). The presence of an impaired GDS or AIS scale scores did not relate to differences in the participants’ scores in any of the assessments. There was not significant association between the score of depressive symptoms (GDS) at baseline or at follow-up and PSA level (*p* = 0.17 and *p* = 0.81, respectively). There was not significant association between the score of depressive symptoms (GDS) at baseline or at follow-up and Gleason score (*p* = 0.26 and *p* = 0.59, respectively). There was no significant association between the score of depressive symptoms (GDS) at baseline or at follow-up and TNM stages (*p* = 0.24 and *p* = 0.42, respectively). There was no significant association between the score of sleep quality scale (AIS) at baseline or at follow-up and PSA level (*p* = 0.31 and *p* = 0.65, respectively). There was not significant association between the score of sleep quality scale (AIS) at baseline or at follow-up and Gleason score (*p* = 0.53 and *p* = 0.69, respectively). There was not significant association between the score of sleep quality scale (AIS) at baseline or at follow-up and TNM stages (*p* = 0.66 and *p* = 0.83, respectively).

Correlations were calculated to determine the interaction between the quantitative variables. Statistically significant correlations were found between the age and the baseline and the final scores of the two cognitive assessments, but not for the sleep disturbances or the depressive symptoms. Correlations were also found between the two cognitive tests’ baseline and follow-up scores, both internal and crossed. These are shown in Table 3 and Table 4. Neither the AIS nor the GDS scores showed correlations between them nor with any of the cognitive assessments.

Partial correlations were also calculated. When these correlations were controlled by age group and education level, some tests and scales’ initial and final assessments showed statistically significant correlations (BCog, AIS and GDS). Moreover, a statistically significant correlation was found between the final score of the BCog and the GDS scale. These correlations are also shown in Table 3 and Table 4.

The internal consistency and the internal correlations of the BCog were calculated in the two assessments. In the baseline assessment, the BCog Cronbach’s alpha was 0.78, and 0.77 in the follow-up.

## 4. Discussion

In this longitudinal study, after one year of treatment with ADT with luteinizing hormone-releasing hormone (LHRH) analogues, the participants did not show a decline in their cognitive performance. By contrast, men in the youngest group improved their MMSE and BCog scores in the follow-up compared to the baseline assessment. A statistically significant decrease of the sleep quality was found, with more men exhibiting an impaired result of the AIS score. More men presented with an impaired GDS score in the follow-up assessment, but the differences were not statistically significant.

The BCog scores obtained in the two assessments by the youngest group of participants were comparable to those described for the general population. In contrast, the oldest group obtained lower scores than the participants with schizophrenia in which it was also validated [43]. There were differences between baseline and follow-up scores of the MMSE, with a discreet increase in the second assessment in the youngest group of participants. No statistically significant differences were found in the scores of the BCog. However, when considering the participants’ ages, it was found that there was a decrease in the mean scores in older men while in younger men, the contrary happened. According to this data, the study participants did not decrease their cognitive performance, as happened in previous longitudinal studies [48,49,50]. These findings are valuable for clinical decision in men with PCa patients since the pharmacological treatment with LHRH analogues is the first-line treatment for many patients. The safety of their uses at least during one year over cognitive functions suggests it does not implicate any significant concern regarding this type of toxicity, assessed by two different cognitive assessment tools, and this could be important for some active patients. A small cognitive improvement was observed in specific cognitive domains.

A prospective controlled trial by Alibhai et al. assessed eight cognitive domains and found no adverse effects on cognitive function based on 12 months of ADT use in older men with PCa. In a cross-sectional study of 57 patients with non-metastatic PCa and 51 age-matched controls, ADT was not associated with alterations in cognitive function [51]. Another prospective controlled trial compared patients with non-metastatic PCa who initiated continuous ADT, patients with PCa who did not receive ADT, and healthy controls. Twelve months of ADT was found not to be associated with changes in self-reported cognitive concerns [52]. However, the data obtained from patient-reported outcome measures should be considered with caution because, being subjective, they are based on personal perceptions of cognitive function and may be affected by factors such as mood and fatigue. Objective tests remain the gold standard for measuring cognitive function, allowing the identification of treatment-related cognitive problems that can affect daily life. However, objective tests provide a useful measure of patients’ perceptions of impairment and its impact on quality of life [53,54]. A US population-based analysis involving more than 100,000 men came to the same conclusion with information based on self-reported subjective evidence. ADT was not associated with an increased risk of cognitive impairment than patients with PCa who had not received ADT in the general population [55]. A systematic review and a meta-analysis of cognitive impairment in men with PCa receiving ADT also found no statistically significant risk of overall cognitive impairment after ADT [20,56]. As in these studies, we detected no statistically significant decreases of the cognitive performance in the sample, but this finding should be considered cautiously, as the decline of the scores might need a bigger sample size or a longer period to appear. Moreover, the age-associated physiological cognitive deterioration might synergize with the treatment with ADT.

In the present study, the AIS scores revealed a deterioration of the participants’ sleep quality, with more men presenting an impaired result in the second assessment. This finding coincides with some previous research that assessed men with PCa [35,57] and people treated for other cancer types [39]. To the best of our knowledge, the evidence of sleep disorders and the methods to mitigate them in men treated with ADT is limited; in fact, it was not among the general side effects that we advised our patients about until now. The relationship between lower testosterone concentrations and sleep disturbances is well established [58,59,60] and seems to be bidirectional, revealing a reduction of testosterone levels in young men samples with experimental sleep restriction [61,62]. However, some studies obtained different results in young men [63], and testosterone therapy showed a reduction in sleep duration in older men [64]. Men receiving ADT should be recommended to avoid known harms to sleep quality, together with the rest of the recommendations, like sleep hygiene measures or pharmacological treatment.

The GDS scores were higher in the second assessment, implying an increase of the men who had a positive screening for depressive symptoms, but the differences were not statistically significant. This finding contrasts with previous longitudinal studies that found a higher prevalence of depression in men treated with ADT [65,66,67,68,69,70,71,72], and in line with others, that did not confirm such a relationship [73,74]. Earlier studies suggested a relationship between borderline or lower testosterone levels and depressive symptoms in men [75,76,77], especially when the reduction of testosterone concentrations was longitudinal [78]. This longitudinal change might explain the trend of increasing depressive symptoms we observed in the sample studied, but the time interval between the baseline and the follow-up measurements might not have been enough to confirm statistically significant changes in the scores. Previous studies developed in the ageing population have proven the relevance of depressive symptoms in cognitive performance [79].

As previous research had concluded, in our study, men who were younger and had achieved higher education levels showed better cognitive performance [17]. No statistically significant differences were found in the cognitive performance between those who had impaired sleep or depression results and those who did not. Statistically significant correlations appeared between variables when partial correlations controlled by age and education level were calculated. The GDS follow-up score significantly correlated with the BCog score. We also found a correlation between the depressive symptoms and the sleep quality in the baseline assessment, but it was not confirmed in the follow-up.

To manage the potential impact of ADT in men and their partners’ lives, previous studies have highlighted the need for a multidisciplinary approach through psychosocial interventions [38,80], educational interventions [81], and the role of exercise medicine [82,83]. As in the study of the ADT effects, there are significant gaps in the literature regarding the effectiveness of interventions to manage precise adverse effects of ADT, such as sleep deterioration [84]. We did not observe any differences regarding the outcomes of the study based on prior prostatectomy. However, we should be cautious about these results since the impact of general anaesthesia required for major surgery such as prostatectomy on cognitive impairment is controversial and complex [85,86]. Several studies have shown an association between exposure to surgery under general anaesthesia and the development of delayed neurocognitive recovery in only a subset of patients [85,87]. There are conflicting data on the relationship between exposure to anaesthesia and the development of long-term neurocognitive disorders, or the development of dementia in the patient population with normal preoperative cognitive function. Among patients, a prior prostatectomy was associated with impaired immediate and delayed verbal memory in one study [88], and a detailed analysis of different type of cognitive domains is required in longer follow-up studies in order to shed some light on this relevant issue.

This study has some strengths, as its longitudinal design. In this study, as suggested in previous studies [84,89], the cognitive assessment in men with PCa has been supplement with other factors, like mood, age, education levels. Moreover, two different brief cognitive batteries were used, allowing the individual analysis of the specific cognitive functions, not only considering cognition as a whole. The scales used to measure depressive symptoms (GDS), and sleep disturbances (AIS) were validated for older men.

This study does have some limitations, too. The sample size was too small to infer about the statistical comparison between groups based on socio-demographic data and clinical findings. There were some heterogeneities in the prostate cancer burden such as patients previously having submitted to prostatectomy versus non prostatectomy group or metastatic versus non-metastatic prostate cancer which could have limited the power of the difference analysis between groups. The sample was heterogeneous in age and education level, but these variables were considered confounding factors in the statistical analyses. Moreover, our findings’ comparability may be limited due to the use of screening tests or batteries. The BCog scale was validated in a younger sample, but it showed acceptable results to measure cognition in older men, with adequate internal consistency in the baseline and the follow-up assessment. Even though we did not detect cognitive impairment by ADT in our series, it is crucial to take into account the possibility that some individuals with cognitive impairment present before ADT may suffer a worsening of their cognitive impairment or that studies with a follow-up longer than one-year could detect cognitive deficits under ADT in individuals with PCa. A further large-numbered study design should be required to support the conclusions of the study.

## 5. Conclusions

In this study, we did not find evidence of the relationship between ADT (under LHRH analogues) and cognitive deterioration in men with PCa despite using two different cognitive tests. Younger age and higher education level were correlated to higher scores in the cognitive tests. When controlled for age and education level, the follow-up scores of the BCog were found to be correlated to the depressive symptoms and to the sleep quality. Our results suggest changes in sleep quality in men with PCa undergoing ADT and a potential increase in depressive symptoms. We found a correlation between depressive symptoms and sleep quality. It is necessary to inform patients before the beginning of the treatment and adopt preventive measures to preserve their quality of life. More research is still needed.

## Figures and Tables

**Table 1:** Sociodemographic and clinical characteristics of the participants.

Variable	Values	Frequency (%); Mean (±SD)	
Age	Under 75	21 (63.6%)	70.8 (±9.8)
	75 or more	12 (36.4%)	
Education level completed	None	6 (18.2%)	
	Primary studies	13 (39.4%)	
	Secondary studies	8 (24.2%)	
	University studies	6 (18.2%)	
Marital status	Married or in a relationship	29 (87.9%)	
	Divorced	3 (9.1%)	
	Other	1 (3%)	
Employment status	Retired	27 (81.8%)	
	Working	3 (9.1%)	
	Other	3 (9.1%)	
Form of cohabitation	Alone	3 (9.1%)	
	With his wife or partner	24 (72.7%)	
	With his family	6 (18.2%)	
TNM stage	II	19 (57.6%)	
	III	7 (21.2%)	
	IV	7 (21.2%)	
Metastases	No	26 (78.8%)	
	Yes	7 (21.2%)	
Prostatectomy	No	11 (33.3%)	
	Yes	22 (66.6%)	
PSA level at enrollment of the study		1.86 (±2.5)	
Gleason score		7.1 (±1.0)	
ADT Drug	Leuprorelin	9 (27.3%)	
	Triptorelin	24 (72.7%)	

SD: Standard deviation; TNM: Tumor, nodes, metastases; PSA: Prostate-specific antigen; ADT: Androgen-deprivation therapy.

**Table 2:** Evolution of the cognitive performance, sleep disturbances and depressive symptoms over one year of treatment.

		Initial Assessment Median (IQR); Frequency (%); Mean (SD)	Follow-up Assessment Median (IQR); Frequency (%); Mean (SD)	*p*-Value
AIS	Score	2 (1–3.5)	4 (1–7.5)	*p* = 0.018 *
	Under 75 years old	2 (1–4.5)	3 (0.5–7)	0.51
	75 years old or more	2 (0–2.75)	5.5 (2.5–7.75)	0.009 *
	Normal	28 (84.8%)	21 (63.6%)	*p* = 0.001 *
	Impaired	5 (15.2%)	12 (34.4%)	
GDS	Score	2 (1–3.8)	2 (1–6)	*p* = 0.194
	Under 75 years old	2 (1–4)	1 (0.5–5.5)	0.77
	75 years old or more	1.5 (0.25–2.75)	3.5 (1.25–7)	0.074
	Normal	30 (90.9%)	23 (69.7%)	*p* = 0.164
	Impaired	3 (9.1%)	10 (30.3%)	
MMSE	Total score	28 (25–30)	29 (27–30)	*p* = 0.035 *
	Under 75 years old	30 (26.5–30)	29 (28–30)	*p* = 0.21
	75 years old or more	25.5 (25–28)	27 (26–29.75)	*p* = 0.046 *
	Orientation	5 (5–5)	5 (5–5)	*p* = 0.058
	Spatial orientation	5 (5–5)	5 (5–5)	*p* = 0.317
	Registration	3 (3–3)	3 (3–3)	*p* = 0.317
	Attention and calculation	5 (4–5)	5 (5–5)	*p* = 0.168
	Recall	3 (2–3)	3 (2–3)	*p* = 0.465
	Language	9 (7–9)	9 (8–9)	*p* = 0.460
BCog	Total score	68.6 (±14.5)	69 (±13)	*p* = 0.857
	Under 75 years old	74.14 (±12.78)	75.50 (± 11.29)	*p* < 0.001 *
	75 years old or more	58.96 (±12.28)	57.50(±6.23)	*p* = 0.79
	Communication	10 (7.9–12.5)	9 (6–11.5)	*p* = 0.177
	Attention	11 (10–12)	11 (10.5–12)	*p* = 0.550
	Recent memory	6 (5–7)	6 (5–7)	*p* = 0.717
	Concentration	4 (3–5)	3 (3–4.5)	*p* = 0.582
	Remote memory	20.4 (±7.3)	20.4 (± 7.1)	*p* = 0.979
	Orientation	8 (8–8)	8 (8–8)	*p* = 0.979
	Calculation	4.2 (±2.3)	5.2 (±2.2)	*p* = 0.001 *
	Executive function	6 (4–7.5)	6 (4–7)	*p* = 0.333

SD: Standard deviation; IQR: Interquartile range; AIS: Athens Insomnia Scale; GDS: Geriatric Depression Scale; MMSE: Mini-Mental State Examination; BCog: Brief Scale for Cognitive Evaluation. The *p*-values were calculated through different statistical tests. T-tests were used for variables that adapted the normal distribution. Wilcoxon ranks tests were used for variables that did not adapt to the normal distribution.

**Table 3:** Correlations among quantitative variables (baseline assessment).

	Age 1st	MMSE 1st	BCog 1st	AIS 1st	GDS 1st
Age 1st		−0.39 *	−0.54 **	−0.16	−0.29
MMSE 1st	−0.39 *		0.72 ** 0.53 **	−0.13 −*0.11*	0.2 *0.12*
BCog 1st	−0.54 **	0.72 ** 0.53 **		−0.17 −*0.21*	0.11 −*0.07*
AIS 1st	−0.16	−0.13 −*0.11*	−0.17 −*0.21*		0.3 0.53 **
GDS 1st	−0.29	0.2 *0.12*	0.11 −*0.07*	0.3 0.53 **	

* The correlations were statistically significant *p* < 0.05, ** The correlations were statistically significant *p* < 0.01, The correlations controlled by age and education level are shown in italics. MMSE: Mini-Mental State Examination; BCog: Brief Scale for Cognitive Evaluation; AIS: Athens Insomnia Scale; GDS: Geriatric Depression Scale.

**Table 4:** Correlations among quantitative variables (follow-up assessment).

	Age 2nd	MMSE 2nd	BCog 2nd	AIS 2nd	GDS 2nd
Age 2nd		−0.44 *	−0.76 **	0.16	−0.04
MMSE 2nd	−0.44 *		0.48 ** 0.16	0.22 *0.35*	0.15 *0.34*
BCog 2nd	−0.76 **	0.48 ** 0.16		−0.12 *0.02*	0.13 0.37*
AIS 2nd	0.16	0.22 *0.35*	−0.12 *0.02*		0.29 *0.29*
GDS 2nd	−0.04	0.15 *0.34*	0.13 0.37 *	0.29 *0.29*	

* The correlations were statistically significant *p* < 0.05, ** The correlations were statistically significant *p* < 0.01, The correlations controlled by age and education level are shown in italics. MMSE: Mini-Mental State Examination; BCog: Brief Scale for Cognitive Evaluation; AIS: Athens Insomnia Scale; GDS: Geriatric Depression Scale.

## Data Availability

The data presented in this study are available on request from the corresponding author.
